# Human Passage of *Schistosoma incognitum*, Tamil Nadu, India, and Review of Autochthonous Schistosomiasis, South Asia

**DOI:** 10.3201/eid3006.231641

**Published:** 2024-06

**Authors:** Sitara S.R. Ajjampur, Rajiv Sarkar, Richard S. Bradbury

**Affiliations:** Christian Medical College, Vellore, India (S.S.R. Ajjampur);; Indian Institute of Public Health Shillong, Meghalaya, India (R. Sarkar);; James Cook University, Townsville, Queensland, Australia (R.S. Bradbury)

**Keywords:** Schistosomiasis, Schistosoma, parasites, zoonoses, India, Nepal

## Abstract

A fecal survey in Tamil Nadu, India, revealed 2 persons passed schistosome eggs, later identified as *Schistosoma incognitum*, a parasite of pigs, dogs, and rats. We investigated those cases and reviewed autochthonous schistosomiasis cases from India and Nepal. Whether the 2 new cases represent true infection or spurious passage is undetermined.

In 1926, A.C. Chandler described “a new schistosome infection of man” based on the presence of distinctive terminal spined schistosome eggs from 2 human fecal samples collected in Krishnanagar, West Bengal, and Kalimpong, Sikkim, both in northeast India (*1*). Obtaining fecal samples directly from humans was difficult; thus, both specimens were collected from areas where humans regularly defecated, and the provenance of the specimens could not be confirmed (*1*). No subsequent human infections with that schistosome species, *Schistosoma incognitum*, were reported, and pigs, which are natural hosts, were prevalent in the areas where the samples were collected. Other researchers later considered those 2 infections likely were derived from misidentified pig feces (*2*). However, Chandler claimed in the original report that “from the nature of the stool there was no reasonable doubt that it was human stool” based upon its fresh collection from an area frequented by humans for defecation and the presence of *Trichuris* and *Ascaris* eggs in 1 of the stool samples and hookworm eggs in both samples (*1*). No further reports of *S. incognitum* in human stool were made after Chandler’s initial findings. We report detection of *S. incognitum* from 2 persons in Tamil Nadu, India, and review autochthonous schistosomiasis cases from Nepal and India.

## The Study

In September 2016, as part of a community-based study on hookworm, stool surveys from ≈8,600 participants were conducted in 45 villages in Thiruvanamalai District, Tamil Nadu, India (*3*), an area that has high rates of open defecation. Participants provided written informed consent, and the study was reviewed and approved by the institutional review board of Christian Medical College, Vellore, India (approval no. 8264, 2023 March 27). 

**Figure 1:**
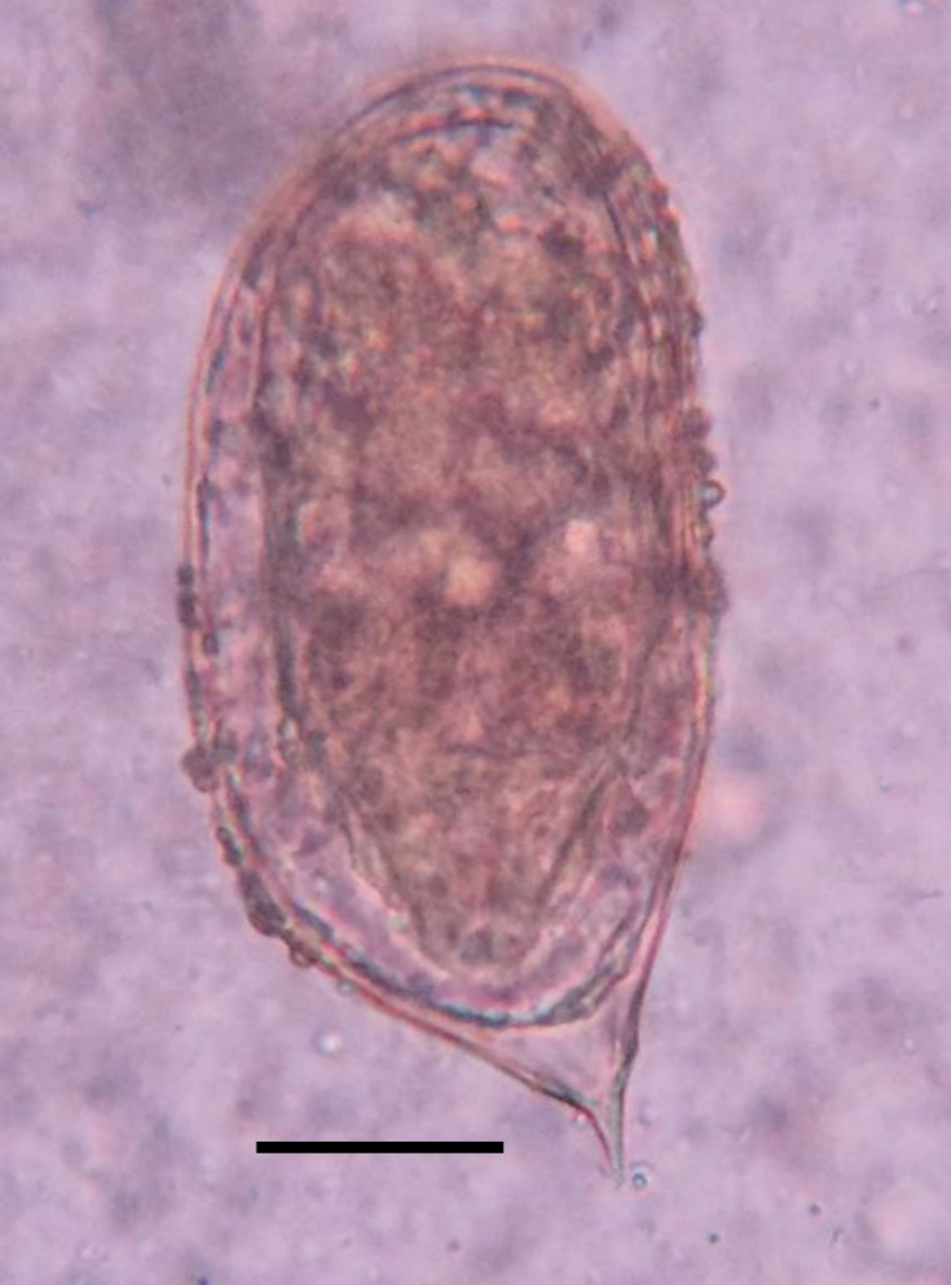
*Schistosoma incognitum* egg identified in the feces of a woman from Tamil Nadu, India. Saline direct smear. Original magnification ×400; scale bar indicates 25 µm.

As part of that study, suspected schistosome eggs were seen in direct wet mounts (2–3 ova/slide) from fecal samples of 2 women, 50 and 35 years of age, who were from the same household. The eggs were 110–120 µm in length, suboval, flatter on one side, and bluntly rounded at the aspinous end and displayed a prominent asymmetric terminal spine (Figure 1). Motile miracidia were clearly visible within the eggs. The eggs were consistent with morphologic descriptions and illustrations of *S. incognitum* (*1*,*2*,*4*).

Both women were generally in good health. The older woman was co-infected with hookworm. Both women belonged to low socioeconomic strata, had no toilet access, and used water from a public tap for drinking and household use. Other infection risks were owning or exposure to animals, including dogs, pigs, and free roaming cattle and poultry, as well as handling animal manure daily. The women’s house was located next to a stream that was used for open defecation. On re-evaluation in 2018, both women were in good health; stool sample examinations, complete blood counts, and liver function tests were within normal ranges, and no *Schistosoma* ova were detected in stool samples. No treatment was given during either observation; the women were advised regarding good hygiene practices.

Those 2 cases likely represent spurious fecal passage of *S. incognitum* eggs after consumption of animal liver, food contaminated with animal feces, or other environmental ingestion. However, long-term or transient true infections cannot be excluded. Species identification relied on morphology of the passed eggs because molecular identification was not possible. 

*S. incognitum* is a natural parasite of pigs, dogs, sheep, goats, and rodents in Asia (*2*,*4*,*5*). In ricefield rats (*Rattus argentiventer*), *S. incognitum* eggs invade the liver and cause hepatic granulomas; less commonly, ectopic egg granulomas can be found in the intestine, stomach, pancreas, or lungs (*5*). *Lymnaea luteola* water snails are the intermediate host (*4*,*6*,*7*).

Experimental infection of primates with *S. incognitum* is possible (*6*,*7*), indicating that human infection also might occur. In 1 experiment, 13 immunocompetent rhesus monkeys (*Macaca mulatta*) were percutaneously exposed to 1,000–2,500 cercariae (*6*). Fatal infection developed in 2 monkeys. At necropsy, 1 monkey harbored 100 mature *S. incognitum* flukes in the intrahepatic portal veins and 4 adult flukes (2 female and 2 male) in the mesenteric veins. Both viable and immature *S. incognitum* eggs were found in the liver but none in the intestinal wall or feces. The second deceased monkey was infected with 400 immature *S. incognitum* flukes in the intrahepatic vessels and 8 in the spleen. Another 4 infected monkeys had 1–6 immature *S. incognitum* flukes in the intrahepatic portal veins, and the other animals were refractory to infection, including 1 monkey exposed multiple times (*6*). 

In another experiment, 10 immunocompetent rhesus monkeys were percutaneously exposed to 2,000–2,500 cercariae (*7*). Upon necropsy at 21–35 days postinfection, 2–114 adult *S. incognitum* flukes were recovered from the hepatointestinal circulation; 2 monkeys also had 2­–3 adult flukes in the lungs. Four monkeys were euthanized at 45–100 days postinfection; 2 were infected with single immature *S. incognitum* flukes (*7*). That study did not state whether the animals were euthanized later because they did not show evidence of infection. Two other studies either found rhesus monkeys were refractory to or only capable of maintaining transient *S. incognitum* infections (*7*). Neither study reported *S. incognitum* eggs in feces from infected monkeys.

**Figure 2:**
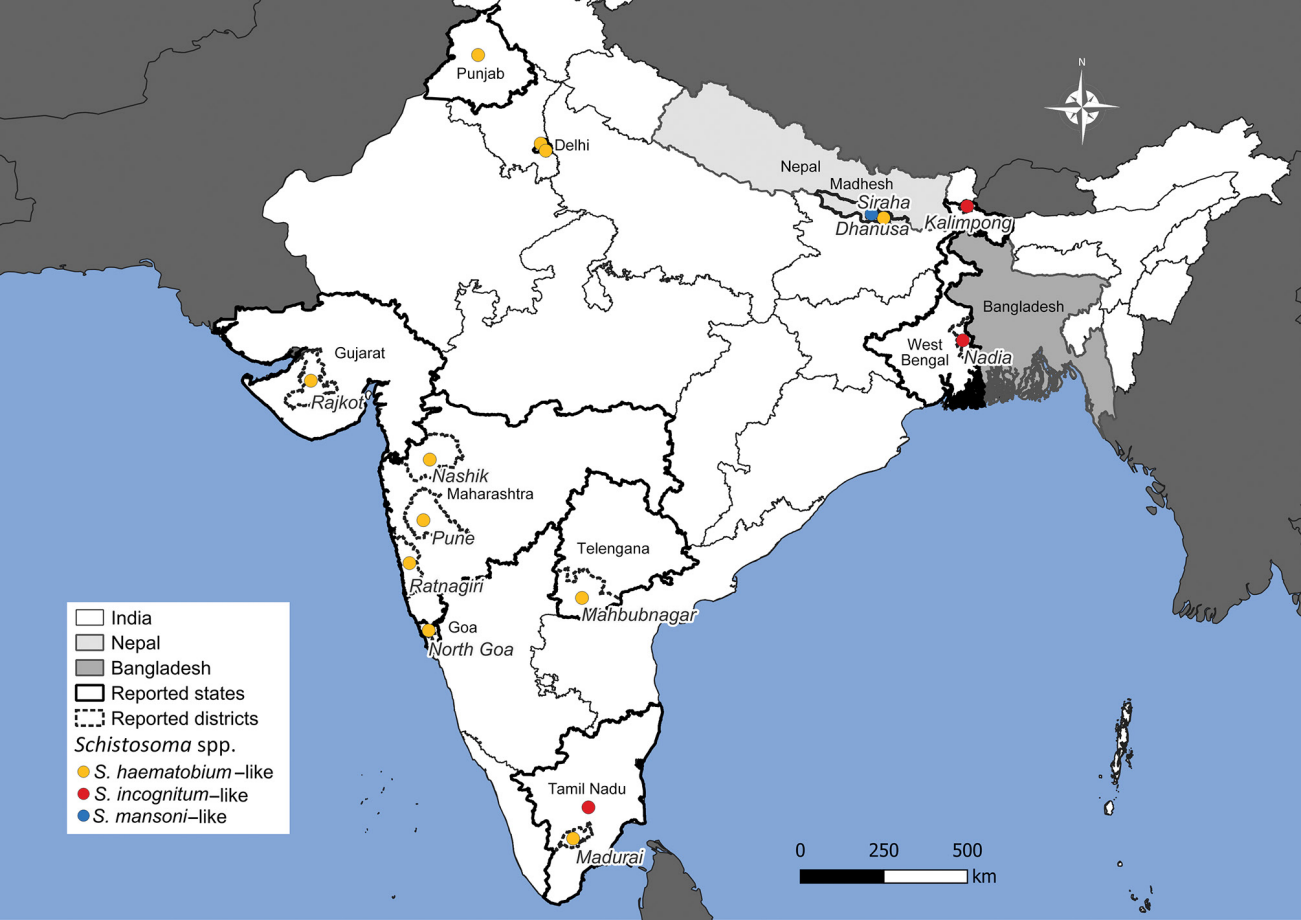
Geographic locations of *Schistosoma incognitum* passage from humans and autochthonous human schistosomiasis in India and Nepal. Map shows credible reports of urinary or fecal passage of schistosome eggs in India and Nepal from this study and others (*1*,*8**–**11*,*14*,*15*). Included patients had no reported travel history to known endemic areas. Where possible, state and district information are provided.

Our report on human passage of *S. incognitum* eggs and that by Chandler (*1*) are not the only reports of schistosomiasis from the subcontinent of India. Many convincing reports from India and Nepal document autochthonous schistosomiasis cases without a history of travel to endemic regions (*8*–*15*) (Figure 2). In India, in 1952, a large focus of genitourinary schistosomiasis, assigned to *S. haematobium*, was discovered in Gimvi Village, Ratnagiri District, Maharashtra (*8*). Of 1,200 village inhabitants, 250 cases were detected, a 21% overall prevalence (*8*). In 1956, *S. haematobium*–like eggs were reported in the feces of 3 people from New Delhi and 1 from Punjab, none with travel histories (*9*). Later, 30% of 3,000 inhabitants of Tirupparankundram Village, Madurai District, Madras, were found to be passing eggs resembling *S. haematobium* (*10*). A 1989 parasitologic survey of Dokur Village, Andhra Pradesh, found samples from 4 participants, 2 stool and 2 urine samples, contained eggs clearly resembling those of *S. haematobium* (*11*), but the 2 positive fecal samples were assumed to have been contaminated by urine during collection (*12*). No travel to *S. haematobium*–endemic regions was reported in those cases (*8*–*12*). Several other reports of schistosomiasis from India lack sufficient evidence or have travel histories to known endemic regions (*12*). Other reports clearly show artifacts mistaken for schistosome eggs (*13*).

Autochthonous schistosomiasis has also been reported from Nepal. In the late 1990s, eggs morphologically indistinguishable from *S. mansoni* were found in the feces of 3 persons from the Dhanusha district, none of whom had traveled outside of Nepal (*14*). A subsequent serologic survey using a *Schistosoma* antibody ELISA assay revealed a seroprevalence of 18.1% in 518 participants from 4 villages (*14*). In 2019, urinary passage of terminal spined schistosome eggs morphologically indistinguishable from *S. haematobium* was reported in a patient from the Siraha district of Nepal with no travel history outside of India and Nepal (*15*).

## Conclusions

Whether reports of human schistosomiasis represent true *S. haematobium*, *S. mansoni*, and *S. incognitum* infections, hybrids of human and animal schistosomes, spurious passage of animal schistosome eggs, or hitherto unrecognized zoonotic schistosome infections remain unclear. Those reports also could represent minor transmission foci after local introduction of schistosomiasis to an area by a traveler or returning resident. We recommend further investigation of the many reports of human schistosomiasis in India and Nepal. Those investigations should include molecular typing and phylogenetic placement to taxonomically identify *Schistosoma* species and surveillance to determine species distribution in the region.
